# Non-strategic detection of identity-threatening information: Epistemic validation and identity defense may share a common cognitive basis

**DOI:** 10.1371/journal.pone.0261535

**Published:** 2022-01-13

**Authors:** Johanna Abendroth, Peter Nauroth, Tobias Richter, Mario Gollwitzer

**Affiliations:** 1 Department of Psychology, University of Würzburg, Würzburg, Germany; 2 Department of Psychology, Philipps-University Marburg, Marburg, Germany; 3 Ludwig-Maximilians-Universität München, München, Germany; University of Hradec Kralove: Univerzita Hradec Kralove, CZECH REPUBLIC

## Abstract

Readers use prior knowledge to evaluate the validity of statements and detect false information without effort and strategic control. The present study expands this research by exploring whether people also non-strategically detect information that threatens their social identity. Participants (*N* = 77) completed a task in which they had to respond to a “True” or “False” probe after reading true, false, identity-threatening, or non-threatening sentences. Replicating previous studies, participants reacted more slowly to a positive probe (“True”) after reading false (vs. true) sentences. Notably, participants also reacted more slowly to a positive probe after reading identity-threatening (vs. non-threatening) sentences. These results provide first evidence that identity-threatening information, just as false information, is detected at a very early stage of information processing and lends support to the notion of a routine, non-strategic identity-defense mechanism.

## Introduction

“*Nothing is easier than self-deceit*. *For what each man wishes*, *that he also believes to be true*.”

                                                                       Demosthenes (349 B.C. [1])

As Demosthenes already observed more than two thousand years ago, humans possess a host of strategies that help them maintain a positive self-concept. Psychological research on motivated reasoning [2] demonstrated that humans are prone to ignore [3], devalue [4], or simply forget [5] information that explicitly or implicitly threatens their identity. Generally, threat detection is assumed to be based on detecting an “is-ought discrepancy” [p. 194, 6]–that is, a discrepancy between a given or anticipated outcome and a preferred outcome. Discrepancy detection therefore constitutes the starting point of most models of psychological threat [7]. The present research looks at the cognitive processes underlying the detection of an identity-related discrepancy (i.e., identity threat). More precisely, we investigate at which stage of information processing identity-threatening information is detected–parallel to comprehension or successively after comprehension. Knowing when identity-threatening information is detected and whether this is an active, deliberate or rather a passive, automatic process is particularly important for all theories concerned about the understanding of how humans cope with and react to threatening information [7–10]. If threatening information is detected non-strategically, downstream psychological reactions may also be triggered non-consciously. On the contrary, if detecting threatening information requires effort and resources, humans may have deliberate control about their defense reactions.

### Epistemic reactions to identity threatening information

Humans tend to reject identity-threatening information regardless of its validity [2]. Countless studies have found that people question the validity of an information (or the credibility of its source) when that information is inconsistent with their prior beliefs, their ideological values, or their self-concept. In their seminal study, Lord, Ross, and Lepper found that proponents (opponents) of the death penalty perceived information questioning (confirming) the deterrent effect of the death penalty as inaccurate and flawed [4]. This research result was further replicated and extended with the finding that individuals reject belief-inconsistent information because it is threatening for their self-concept [11]. Recent research additionally suggests that individuals also reject information as flawed when it implies a threat to their social identity [12, 13]. The rejection of identity-threatening information has been documented in many other research contexts [14–16], all indicating that humans are prone to perceive identity-threatening information as false information. This line of research suggests for example in the field of political decision making [16] that humans have a tendency to more readily accept the validity of information that they want to believe—a phenomenon that has been referred to as motivated reasoning [2]. One intriguing question in this regard is whether identity-threatening information is actually detected as “false” in very early stages of cognitive processes or, by contrast, if labeling identity-threatening information as “false” is a strategic and, thus, more effortful coping response to the identity threat implied by that information. If our cognitive system treats identity-threatening information as false information, an identity threat should be detected at the same level and in an equally non-strategic way as any other false information (similar to the more general confirmation bias, [17]).

Theoretically, two possibilities of the time course of analyzing the validity of information are plausible and can be found in the literature [18]. We will refer to these two possibilities as the “serial model” and the “parallel model” and elaborate on those next.

### Serial model

The “serial model” proposes that individuals should detect a psychological threat only in a second stage of processing, after elaborating on the consequences of the threatening information for the self. Hence, according to the serial model, our cognitive system has first to decode and comprehend the content of a message before false information is internally tagged as “false” [19]. This model is derived from the dual-stage model of language comprehension and information evaluation [20]. This model assumes that while comprehending an assertion, individuals initially accept all incoming information as being true. Evaluating the validity of the information (validation) occurs at a later stage when initially accepted information can be actively rejected. The validation process occurring at the successive stage is assumed to be cognitively demanding, implying that individuals need to have the capacity to engage in validation. Importantly, it is also strategic and optional, implying that individuals engage in such processes only if they are motivated to do so. Hence, the serial model implies an effortful, strategic, and optional stage of evaluating information after initially accepting all incoming information as true. The model stems from empirical evidence demonstrating an affirmation bias in learning statements about fictitious facts that were either denoted as true or false (e.g., “A suffa is a cloud”; [19]). When participants were put under cognitive load or time pressure during learning these statements, they incorrectly remembered “false” statements as true. Thus, according to the dual-stage model, the evaluation of information as identity-threatening is detected only at an optional and strategic second stage of information processing. Given that the evaluative processes at this stage are cognitively costly, individuals can only be expected to engage in these processes if they pursue a specific goal that necessitates the rejection of threatening information.

It is important to point out that one crucial difference between fictitious and identity-threatening information is the amount of background knowledge individuals have available for an evaluation of the statements. For fictitious statements, such as those used by Gilbert and colleagues [19], it is unlikely that individuals had relevant background knowledge to validate the statements. For identity-threatening information, such background knowledge is very likely to exist.

### Parallel model

In contrast to the serial model, the parallel model proposes that identity-threatening information is already detected during encoding. In the psychology of language, it is now widely accepted that language processing proceeds incrementally, with different types of information processed and integrated in parallel when they become available. This view is corroborated by many studies that support the assumption of parallel rather than serial processing for a wide range of psycholinguistic phenomena, from word recognition to perspective taking in discourse [21–23]. In much the same vein, recent research indicates that comprehension and validation (i.e., evaluating information with regard to its validity) of linguistic information are closely intertwined and occur in parallel rather than in successive steps [18, 24, 25]. If this was true, identity-threatening information should already be detected early, that is, parallel to encoding a message. In other words, the parallel model assumes that false information is detected parallel to comprehending the information, leading immediately to false information being flagged as “false.” This model is based on research showing that the validation of incoming information can occur automatically during comprehension, that is, independent of an effortful and strategic successive processing stage [25]. For instance, Richter, Schroeder, and Wöhrmann [26] demonstrated that individuals reject false assertions (such as “Soft soap is edible”) as efficiently as they verified true assertions (such as “Perfume contains scents”), even under additional cognitive load. Richter and colleagues argued that an automatic validation process protects mental representations from being contaminated by false or inaccurate information, thereby enabling an efficient rejection of factually false assertions.

Isberner and Richter [27, 28] provided further evidence that validation is a routine and non-strategic process concerning factual knowledge. In the Stroop-like paradigm used, participants were provided with either a false or true assertion (e.g., assertion: “Soft soap is edible”). However, participants’ task was unrelated to the validity of the assertion. Rather, participants worked on a non-evaluative simple probe task in which they had to react as quickly as possible to a “True”/”False” probe stimulus by pressing one of two different keys (i.e., the key “k” if the word “True” appeared on the screen). Hence, this experimental task does not require or encourage validation, since the reaction (positive [“True”] or negative [“False”] response) is independent of the presented assertion (see also [29]). Isberner and Richter predicted that if validation occurs routinely (i.e., automatically) during comprehension, a negative response tendency should occur for false assertions. This means that it should be harder to provide a positive response (i.e., pressing “k” for “True”) after reading false information because the validation process interferes with the positive responses in the task and thus, slows down response times. Supporting their reasoning, task-irrelevant validity of the statements differentially affected response latencies, resulting in a significant interaction of validity and required response. After reading invalid assertions, positive responses were much slower compared to reading valid assertions. In the meantime, similar epistemic Stroop effects have been demonstrated for different types of information, including audio-visual information [30] and opinion statements that were consistent or inconsistent with participants beliefs [31]. This research suggests that false, implausible, or belief-inconsistent information elicit a negative response tendency interfering with any kind of positive response.

Theoretical accounts of validation and its relationship to language and text comprehension emphasize that validation serves the function to detect false, implausible, or belief-inconsistent information, allowing individuals to reject such information and construe a mental model that is internally consistent and fits with their knowledge and beliefs [32, 33]. In that sense, validation may be construed as a basic (although not perfect) mechanism of epistemic vigilance [34, 35]. In the present context, it is important to note that the emphasis on the rejection of false or implausible information implies that the processing of such information should interfere with positive responses, but not so much that the processing of true or plausible information interferes with negative responses. In line with these assumptions, most studies based on the epistemic Stroop paradigm found interference effects for positive responses in the presence of false, implausible, or belief-inconsistent information, but not for negative responses in the presence of true, plausible, or belief-consistent information (for overviews, see [34, 36]). In the light of the available theory and research, we restrict our hypotheses to interference effects that occur in identity-threatening information combined with positive responses.

### The present research

Similar to easily accessible general world knowledge, individuals already possess all the relevant information to evaluate identity-threatening information. As a result, and in line with the parallel model, we argue that the detection of identity threatening information and its consequential rejection occurs already parallel to the encoding of the information in a passive validation process. Put differently, we postulate an automatic, non-strategic identity protection process that enables the effortless validation of identity-threatening information. Thus, we expect the automatic detection of identity-threatening information to be similar to the automatic detection of false information. To test this assumption, we adopted the paradigm by Isberner and Richter [28] and extended this design such that participants had to react to the probe (“True” vs. “False”) after being presented with (a) false, (b) true, (c) identity-threatening, and (d) identity-non-threatening statements. Replicating Isberner and Richter [28], we expected that, when a reaction to the probe “True” is required, participants react more slowly after false than after true statements. In addition, we expected that participants react more slowly after identity-threatening than after non-threatening statements when a positive response is required. More precisely, the difference in reaction times between true and false statements should be similar to the difference in reaction times between identity-threatening and non-threatening statements.

## Method

Data files and analysis scripts for all analyses reported here and the full experimental materials are available on the OSF (https://doi.org/10.17605/OSF.IO/589W).

### Participants

Seventy-nine native German speaking psychology students participated in the study. Data from two participants, who afterwards admitted that they had not followed the instructions properly, were excluded from the analysis. Therefore, the final sample consisted of 77 participants (79% women, age: *M* = 23.89; *SD* = 3.91 years). Participants provided informed consent at the beginning of the experiment and were reimbursed with 6 € or course credit.

### Material

The stimuli were true, false, threatening, and non-threatening sentences of the structure “[Psychologists] [verb] […],” for example, “Psychologists are sometimes hungry” (the actual stimuli were in German; e.g., “Psycholog(inn)en sind manchmal hungrig”). A norming study (*N* = 10 psychology students) was conducted to select sentences that were threatening vs. non-threatening and true vs. false. Participants in that norming study read 160 sentences and rated whether the sentences were true or false, how certain they were in this judgment (7-point scale ranging from 1 = *very uncertain* to 7 = *very certain*), and to what extent the assertions were threatening to their identity as a psychologist (on a 4-point scale ranging from 1 = *not threatening at all* to 4 = *very threatening*). Certainty ratings were included as a meta-judgmental indicator of belief strength [37]. This norming study allowed for categorizing sentences into threatening (high threat, moderate subjective trueness, and moderate judgment certainty), non-threatening (low threat, moderate subjective trueness, and moderate judgment certainty), true (low threat, high subjective trueness, and high judgment certainty), and false (low threat, low subjective trueness, and high judgment certainty) assertions (for a similar procedure, see [26]).

Based on the results of the norming study, 16 true (e.g., “Psychologists often live in cities”) and 16 false assertions (e.g., “Psychologists do not like coffee”) were selected. On average, the proportion of true-judgments differed significantly between true sentences (93%) and false sentences (2%); *t*(15) = 42.37, *p* < .001, *d* = 28.33. In contrast, certainty ratings (true sentences: *M* = 5.40, *SD* = 0.99; false sentences: *M* = 5.32, *SD* = 0.67; *t*(15) = 0.23, *p* = .81, *d* = 0.10) and threat ratings (true sentences: *M* = 1.51, *SD* = 0.35; false sentences: *M* = 1.49, *SD* = 0.15; *t*(15) = 0.21, *p* = .84, *d* = 0.08) did not differ significantly between the respective types of sentences. In addition, 16 identity-threatening (e.g., “Psychologists are very unpopular”) and 16 non-threatening sentences (e.g., “Psychologists are often altruistic”) were selected. On average, threat judgments differed significantly between threatening sentences (*M* = 2.79, *SD* = 0.48) and non-threatening sentences (*M* = 1.20, *SD* = 0.25); *t*(15) = 13.67, *p* < .001, *d* = 3.21. In contrast, neither the proportion of true judgments (threatening sentences: 44%; non-threatening sentences: 46%; *t*(15) = 0.45, *p* = .66, *d* = 0.15) nor average certainty judgments (threatening sentences: *M* = 4.15, *SD* = 0.35; non-threatening sentences: *M* = 3.99, *SD* = 0.51; *t*(15) = 1.06, *p* = .31, *d* = 0.37) differed between the respective types of sentences. Furthermore, 64 items from the remaining sentences were chosen as filler items. In 48 of these trials “psychologists” was replaced by “physicians.”

### Procedure

**Fig 1:**
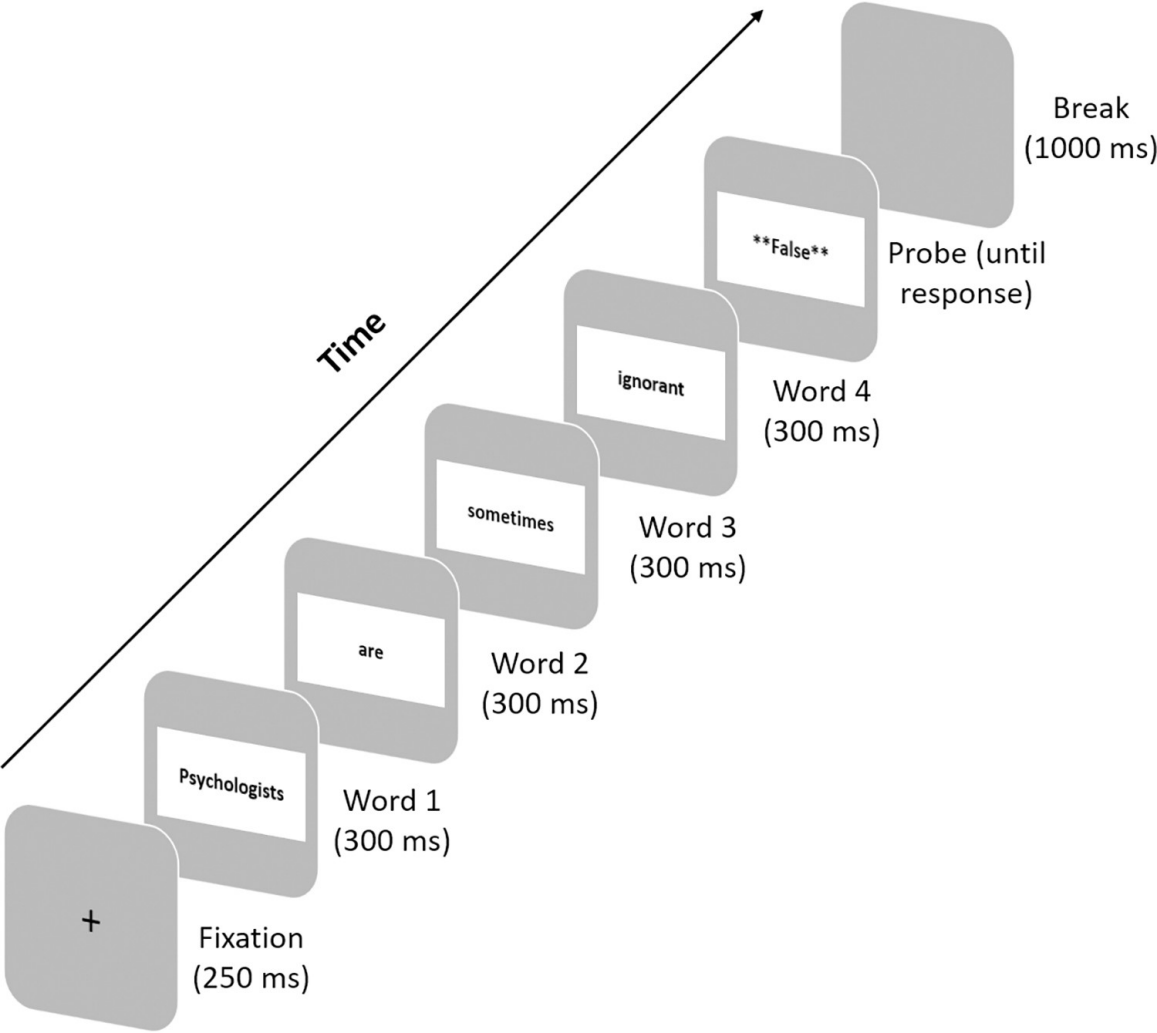
Experimental trial structure.

The procedure closely followed Isberner and Richter [27]. Participants were tested in groups of up to five individuals in a cubicle lab, where they worked individually on computers. After informed consent was obtained (see below), participants were made familiar with the computerized task. They were asked to rest the index fingers of their left and right hand on the two response keys throughout the experiment and to respond as fast and as accurately as possible. All stimuli were presented in an individually randomized order. After every 40 trials, participants were allowed to take a short break. The first eight were practice trials, after which participants had the opportunity to ask questions before starting the actual experiment. Response latencies of the practice trials were not included in the analyses. All stimuli were presented word-by-word on a computer screen using Rapid Serial Visual Presentation with a fixed rate of 300 ms per word; all words were presented in black font (Arial, approximate height 1 cm) against a white background. Every trial was followed by a blank screen presented for 1,000 ms (see Fig 1).

#### Experimental trials

In the 64 experimental trials, the probe “**Richtig**” (“True”) or “**Falsch**” (“False”) appeared after the final word of the stimulus sentence, prompting participants to respond with the corresponding key (“k” for the “True” probe, “d” for the “False” probe). Half of the trials were presented with the “True” probe and the other half with the “False” probe. The probe was presented orthogonally to the condition of the sentence; that is, it was independent of truth and threat of each sentence. Importantly, participants were only required to identify the probe word and press the corresponding key, regardless of proposition of the sentence. The probe remained on the screen until the participant provided a response.

#### Filler trials

In the 64 filler trials, the probe appeared after the second to fifth word (16 trials each) of the sentence to make the appearance of the probe less predictable. Half of the filler trials were presented with the “True” probe, whereas the other half were presented with the “False” probe.

#### Comprehension questions

On half of 64 filler trials, a comprehension question was presented immediately after the sentence, which required comprehension but not validation of the sentence. Specifically, participants were asked to indicate whether the sentence described an action. Participants were informed before the experiment that they would be asked comprehension questions and were instructed to process the content of the sentences to be able to answer these questions. To make sure that participants understood the importance of reading for comprehension before starting the actual experiment, half of the practice trials included comprehension questions.

#### Ethics statement

Before starting the study, informed consent was obtained. Participants read detailed information regarding ethical guidelines (i.e., that the data would be fully anonymized, that participation was voluntary, that they had the right to withdraw their consent to participate at any time, etc.). Only participants who gave their consent could start the study. The study was conducted as part of a research project for which no ethics approval was required, neither from the funding agency nor from the department. According to German laws and ethical regulations for psychological research (see https://www.dgps.de/die-dgps/aufgaben-und-ziele/berufsethische-richtlinien/#c53), gathering IRB approval is not necessary if (i) the data are fully anonymized, (ii) the study does not involve deception, (iii) participants’ rights (e.g., voluntary participation, the right to withdraw their data, etc.) are fully preserved, and (iv) participating in the study is unlikely to cause harm, stress, or negative affect. The present study met all of these criteria; therefore, no IRB approval had to be obtained. All collected information that could have made identification of participants possible were deleted before analysis (i.e., e-mail addresses collected for the raffles).

## Design

The design was a full 4 (condition: threat, no-threat, false, true) × 2 (probe: positive [“True”] vs. negative [”False”]) within-subjects design. The order in which probes (“True” vs. “False”) were combined with a sentence was counterbalanced across participants. Response latencies were recorded as dependent variables. Before the experiment, participants’ social identification with psychology students was measured with an established self-report measure (ranging from 1 to 6; *M* = 3.97, *SD* = 0.55, [38]). An exploratory analysis including participants’ social identification as a covariate did not alter the effects reported here, nor did identification moderate these effects. We therefore refrain from reporting these analyses here.

## Results

### Data analysis

Response latencies were analyzed with a linear mixed model (LMM) with participants and items included as random factors, which implies that participants’ mean reaction times (i.e., individual differences in responsivity) as well as items’ mean reaction times (i.e., difficulty differences between items) were allowed to vary. This type of analysis accounts for the fact that both participants and items represent samples of larger populations. LMM allows for a stringent test of the hypothesized effects of the independent variables in one single model by crossing random effects for participants and items [39]. Probe (true = 1, false = -1) and condition were contrast-coded (contrast 1 “threat validation”: threatening = 1, non-threatening = -1, false = 0, true = 0; contrast 2 “truth validation”: threatening = 0, non-threatening = 0, false = 1, true = -1; contrast 3 “threat vs. truth”: threatening = 1, non-threatening = 1, false = -1, true = -1) and included as fixed effects into the model, complemented by the respective interaction terms. We expected the contrast 1 “threat validation” × probe and the contrast 2 “truth validation” × probe interaction terms to be similar in size and direction: when a positive reaction is required, non-threatening and true assertions should lead to faster responses compared to threatening and false assertions. In addition, the presentation position of each sentence (1 to 128) and the sentence length (4 to 10 words) were included as covariates to control for position and length effects.

The LMM analysis was conducted with the lmer command of the lme4 package for R [40] and *t*-values and degrees of freedom were calculated using lmerTest (which implements Satterthwaite’s approximation; [41]). For the sake of conciseness, only significance tests for the independent variables are reported in the text as these are directly relevant for the hypotheses.

**Fig 2:**
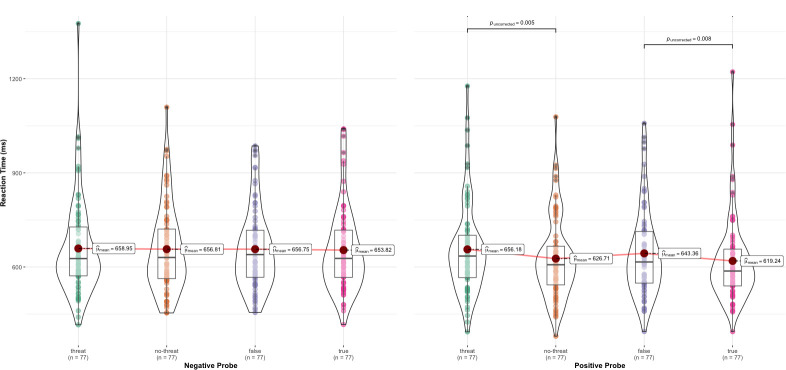
Mean response latency by condition and probe. The figure was computed with the R-package ggstatsplot [42]. Error bars correspond to ±1 standard error of the mean computed for within-subjects designs [43].

Response latencies were calculated for correct responses (97% of the responses in experimental trials) and latencies deviating more than three standard deviations from either the subject or item mean (6.60% of all correct latencies) were treated as outliers and removed from the data set [27]. Fig 2 displays the mean correct response latencies as a function of condition and probe and Table 1 shows the results of the LMM for the fixed effects. According to the parallel model false and threatening assertions should delay positive (but not negative) responses whereas the serial model does not assume an interfering effect of threatening and false assertion on positive responses.

**Table 1:** Results for the fixed effects of the LMM.

	Estimate	*SE*	*Df*	*t*-value	*p*
(Intercept)	647.85	15.02	88	43.12	< .001
Item position	-33.85	2.15	4477	15.77	< .001
Sentence length	-15.12	4.76	59	3.18	0.002
Contrast 1 “threat validation” (threatening vs. non-threatening)	21.57	13.37	59	1.61	0.112
Contrast 2 “truth validation” (true vs. false)	14.52	13.19	58	1.10	0.275
Contrast 3 “threat vs. truth” (threatening & non-threatening vs. true & false)	17.67	18.75	59	0.94	0.350
Probe	21.17	4.21	4466	5.02	< .001
Contrast 1 “threat validation” × probe	-25.12	11.93	4467	2.11	0.035
Contrast 2 “truth validation” × probe	-23.73	11.90	4466	1.99	0.046
Contrast 3 “threat vs. truth” × probe	-11.70	16.85	4466	0.69	0.488

*N*_participants_ = 77, *N*_items_ = 64.

Participants reacted quicker to a positive probe (*M* = 633, *SD* = 192) than to a negative probe (*M* = 653, *SD* = 193), *t*(4466) = 5.02, *p* < .001.However, this effect was qualified by the interactions terms contrast 1 “threat validation” × probe, *t*(4467) = 2.11, *p* = .035, and contrast 2 “truth validation” × probe, *t*(4466) = 1.99, *p* = .046. Simple main effects analysis revealed, in line with the predictions of the parallel model, that positive responses were faster after non-threatening sentences (*M =* 623, *SD* = 177) than after threatening sentences (*M* = 653, *SD* = 205), *t*(76) = 2.86, *p* = .005, *d* = 0.32, 95% CI [0.09, 0.55]. Similarly, and replicating Isberner and Richter [28], positive responses were faster after true sentences (*M* = 615, *SD* = 190) than after false sentences (*M* = 639, *SD* = 192), *t*(76) = 2.71, *p* = .008, *d* = 0.31, 95% CI [0.08, 0.53]. Additionally, we tested whether these simple effects differ in size by testing the difference of the threatening minus the non-threatening sentences of positive responses (*M* = 29, *SD* = 90) against the difference of the false minus the true sentences of positive responses (*M* = 24, *SD* = 78). In line with the parallel model, these effects did not differ significantly, *t*(76) = 0.42, *p* = .67, *d* = 0.05, 95% CI [-0.18, 0.27]. Reacting time differences between false and threatening information were neither significant for positive responses (*t*(76) = 0.32, *p* = .75) nor for negative responses (*t*(76) = 0.24, *p* = .81).

## Discussion

This experiment tested whether identity-threatening information is detected at the earliest possible stage of information processing, namely parallel to the comprehension of the respective information. The results demonstrate that―similar to false information and in line with the parallel model ―identity-threatening information is subject to a very early processing interference. This interference indicates that people non-strategically flag identity-threatening information similar to false information while encoding the information. These results favor the parallel model over the serial model proposing that identity-relevant information is validated parallel to, rather than after, comprehension. These findings contribute to the current literature on threat detection and its psychological consequences by emphasizing the non-strategic nature of cognitive reactions to identity threats [7].

One reasonable interpretation of these results is that, at an early stage of information processing, our cognitive system does not differentiate between identity-threatening and false information and tags threatening information as “false” during comprehension. This interpretation is supported by the fact that the caused interference of identity-threatening information was of similar magnitude as the interference caused by false information. It also resonates with Richter’s proposal that one key task of language validation is to maintain coherent mental representations in general [18]. The present result may have been caused by such a coherence maintenance mechanism that encompasses identity-related constructs. This interpretation is in line with theories assuming a general conflict monitoring mechanism [44] and a general pattern by which individuals detect and react to various kinds of threat [7]. Based on the results reported here, one could speculate that language validation processes detect *any* discrepancies between new information and individuals’ existing mental representations–regardless of whether the information is knowledge- or identity-related. Such a general validation process may be the cognitive basis for the discrepancy detection mechanism proposed by Jonas and colleagues [7] and the general conflict monitoring mechanism proposed by Proulx and colleagues (at least concerning linguistic information) [44]. In that sense, the rejection of identity-threatening information might be viewed as an instance of the more general confirmation bias, that is, the tendency to process information in a way that preserves one’s prior beliefs [17].

However, a valid alternative interpretation is that, even though the interference is similar, the underlying mechanism may be different. Following this interpretation, one system would be responsible for validating factual information and the other for validating identity-relevant information (i.e., threat detection). Both systems would evaluate incoming information with regard to their validity. Future research could investigate whether the observed interferences are caused by one general or two distinct mechanisms.

In sum, our results indicate that when humans are confronted with identity-threatening information, our mental system detects the identity threat at the earliest level of information processing, that is, parallel to encoding the information. Returning to Demosthenes’ observation, it seems that we not only consciously want to believe in information we wish to be true, but that our mental system initially handles undesirable information like false information, immediately dragging us down the road of self-defense.
